# Supplementary material for: A Metastructure Based on Amorphous Carbon for High Efficiency and Selective Solar Absorption
Source: Nanomaterials (Basel). 2024 Mar 27;14(7):580. doi: 10.3390/nano14070580 (PMC11013282; doi:10.3390/nano14070580)
Supplement: Supplementary file 1 [file nanomaterials-14-00580-s001.zip › nanomaterials-2923754-supplementary.pdf]

Supporting Information

A Metastructure Based on Amorphous Carbon for High Efficiency and Selective Solar Absorption

Junli Su[1,2], Gang Chen[1], Chong Ma[1], Qiuyu Zhang[1], Xingyu Li[1,2], Yujia Geng[1,2], Bojie Jia[1], Haihan Luo[1,3]* and Dingquan Liu[1,2,3,]*

[1] Shanghai Key Laboratory of Optical Coatings and Spectral Modulation, Shanghai Institute of Technical Physics, Chinese Academy of Sciences, Shanghai 200083, China; sujl@shanghaitech.edu.cn (J.S.); gangchen@mail.sitp.ac.cn (G.C.); machong@mail.sitp.ac.cn (C.M.); zhangqiuyu@mail.sitp.ac.cn (Q.Z.); lixy12@shanghaitech.edu.cn (X.L.); gengyj2022@shanghaitech.edu.cn (Y.G.); jiabojie22@mail.ucas.ac.cn (B.J.)

[2] School of Physical Science and Technology, ShanghaiTech University, Shanghai 200031, China

[3] School of Optoelectronics, University of Chinese Academy of Sciences, Beijing 100049, China

* Correspondence: haihan.luo@mail.sitp.ac.cn (H.L.); dqliu@mail.sitp.ac.cn (D.L.)

Sample fabrication and heat treatment.

The a-C, Ti@a-C cermet and SiO_2 films were fabricated by using a high-vacuum multi-target magnetron sputtering system (FULL-E, Shanghai, China) at a room temperature (25°C), while commercially available high-purity Ti (≥99.7%, F 3 in), graphite (≥99.999%, F 3 in) and SiO_2 (≥99.99%, F 3 in) were used as target materials, and K9 glass and p-type silicon (100) were used as the substrates. The substrates were first subjected to ultra-sonic washing for 10 mins, successively using mixed solution of alcohol and ether (volume ratio 3:1) and deionized water, then, they were dried with nitrogen gas before being placed in the chamber. In detail, during the co-sputtering process of Ti@a-C, the power of the Ti target was set at 55 W, and the power for a-C was maintained at 300 W. Additionally, the SiO_2 target and a-C protective layer were subjected to RF sputtering at power of 150 W and 300 W, respectively. When the vacuum level of the chamber was pumped to 6.5×10−4 Pa, argon gas with a purity of 99.999% was injected into the chamber. During the deposition process of all films, the growth pressure was controlled by the throttle valve at 0.7Pa (a-C) and 0.6 Pa (Ti@a-C and SiO_2) with argon (Ar) gas flow rate fixed at 100 SCCM.

Ellipsometry analysis

A variable angle spectroscopic ellipsometer (V-VASE, J. A. Woollam, USA) was used to measure and fit the optical constants of Ti, Ti@a-C, a-C and SiO_2 films. The ellipsometric parameters (Δ, Ψ) were obtained in the wavelength range of 300-2500 nm under incident angles of 65, 70 and 75°.The optical constants of a-C and Ti@a-C cermet films were deduced from the ellipsometric parameters fitting process by using dielectric function model of multiple oscillators [1]:

$$\tilde{\varepsilon} = \tilde{\varepsilon}_{\infty} + \tilde{\varepsilon}_{Drude} + \sum_{i} \tilde{\varepsilon}_{Lorentz} + \sum_{j} \tilde{\varepsilon}_{Tauc-Lorentz}$$

Where the Tauc-Lorentz model can well describe the interband transition in the upper half of the conduction band and the absorption phenomenon of the material in the ultraviolet and visible light bands. The measured and fitted ellipsometric parameters were presented in

Figure. S1 As can be seen, the fitting results are in a relatively good agreement with the experimental values when the total fitting MSE less than 10.

The difference between the measurements and the fitting results is defined as the mean square error (MSE):

$$MSE = \frac{1}{2N - M}\sum_{1}^{N}[(\frac{\Psi_i^{mod} - \Psi_i^{exp}}{\sigma_{\Psi,i}^{exp}})^2 + (\frac{\Delta_i^{mod} - \Delta_i^{exp}}{\sigma_{\Delta,i}^{exp}})^2]$$

where Δ and Ψ are the ellipsometric parameters, N is the number of experimental points, M is the number of parameters, σ is the standard deviation, and the superscripts mod and exp refer to the modeled and measured data, respectively.

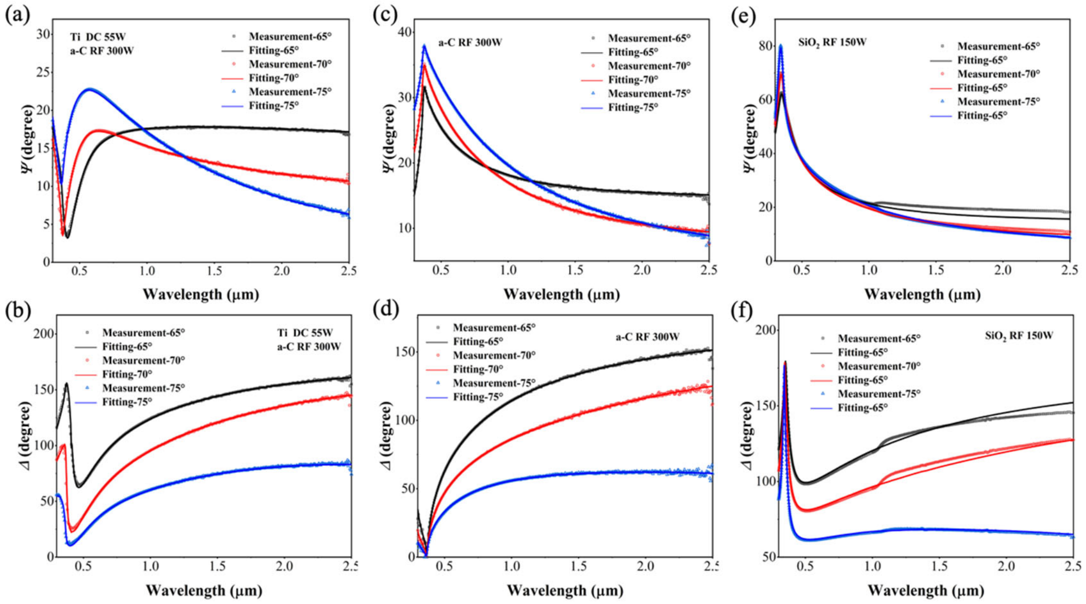

Figure S1. The fitted and measured ellipsometric parameters for: **(a, b)** Ti@a-C cermet film **(c, d)** a-C thin film, **(e, f)** SiO_2 thin film.

References

[1] Sun, B.; Wang, L.; Sun, Y.; Ren, J.; Yang, Y.; Liu, H.; Liang, D.; Li, A.; Wang, C. Optical Performance, Thermal Stability, and Failure Analysis of the WNx-Si3N4 Multilayer Solar Selective Absorbing Coatings. *ACS Applied Energy Materials* **2022**, *5*, 1883-1893, doi:10.1021/acsaem.1c03373.
